# After virus exposure, early bystander naïve CD8 T cell activation relies on NAD^+^ salvage metabolism

**DOI:** 10.3389/fimmu.2022.1047661

**Published:** 2023-02-01

**Authors:** Namit Holay, Barry E. Kennedy, J. Patrick Murphy, Prathyusha Konda, Michael Giacomantonio, Tatjana Brauer-Chapin, Joao A. Paulo, Vishnupriyan Kumar, Youra Kim, Mariam Elaghil, Gary Sisson, Derek Clements, Christopher Richardson, Steven P. Gygi, Shashi Gujar

**Affiliations:** ^1^ Department of Pathology, Dalhousie University, Halifax, NS, Canada; ^2^ IMV Inc, Halifax, NS, Canada; ^3^ Department of Biology, University of Prince Edward Island, Charlottetown, PEI, Canada; ^4^ Department of Microbiology and Immunology, Dalhousie University, Halifax, NS, Canada; ^5^ Dana Farber Cancer Institute, Harvard Medical School, Boston, MA, United States; ^6^ Flow Cytometry Core Facility, Dalhousie University, Halifax, NS, Canada; ^7^ Department of Cell Biology, Harvard Medical School, Harvard University, Boston, MA, United States; ^8^ Department of Microbiology and Immunology, Stanford University, Stanford, CA, United States; ^9^ Canadian Centre for Vaccinology, IWK Health Centre, Goldbloom Pavilion, Halifax, NS, Canada; ^10^ Department of Pediatrics, Dalhousie University, Halifax, NS, Canada; ^11^ Department of Biology, Dalhousie University, Halifax, NS, Canada; ^12^ Beatrice Hunter Cancer Research Institute, Halifax, NS, Canada; ^13^ Cancer Immunotherapy: Innovation & Global Partnerships, Faculty of Medicine, Dalhousie University, Halifax, NS, Canada

**Keywords:** antiviral immunity, CD8 T cells, bystander activation, naïve CD8 T cells, type I interferons, immunometabolism, NAD^+^ salvage metabolism, metabolic reprogramming

## Abstract

CD8 T cells play a central role in antiviral immunity. Type I interferons are among the earliest responders after virus exposure and can cause extensive reprogramming and antigen-independent bystander activation of CD8 T cells. Although bystander activation of pre-existing memory CD8 T cells is known to play an important role in host defense and immunopathology, its impact on naïve CD8 T cells remains underappreciated. Here we report that exposure to reovirus, both *in vitro* or *in vivo*, promotes bystander activation of naïve CD8 T cells within 24 hours and that this distinct subtype of CD8 T cell displays an innate, antiviral, type I interferon sensitized signature. The induction of bystander naïve CD8 T cells is STAT1 dependent and regulated through nicotinamide phosphoribosyl transferase (NAMPT)-mediated enzymatic actions within NAD^+^ salvage metabolic biosynthesis. These findings identify a novel aspect of CD8 T cell activation following virus infection with implications for human health and physiology.

## Introduction

CD8 T cells are an important arm of cellular immunity to viruses and are responsible for identifying and eliminating virus-infected cells during the adaptive phase of the immune response. Following virus exposure, T cells can be activated by T cell receptor (TCR)-dependent and -independent mechanisms. Upon TCR engagement, T cells rapidly lose their naive phenotype, become CD44hi, and are known as differentiated T cells (1). In TCR- independent activation, known as bystander activation, cytokines play an important role in the activation of effector functions in pre-existing memory T cells. Specifically, the cytokines IL-15 and IL-18 can drive antigen independent IFN-γ secretion or cytolytic activity *via* NKG2D in non-specific, pre-existing memory T cells (2, 3). Naïve CD8 T cells can also undergo bystander activation because of their presence in an inflammatory milieu. Some studies have demonstrated that bystander activation during chronic virus exposure can drive naïve CD8 T cells with memory-like phenotype (4) or cause them to differentiate into memory-like T cells upon late priming (5). Interestingly, infections and inflammation driven by the cohousing of laboratory mice with pet store mice can activate naïve CD8 T cells in a bystander manner and impact their homeostasis and function (6). Type I interferons are rapidly produced early in infection in response to viral exposure. Some viruses, such as SARS-CoV2, drive severe disease by dysregulating and delaying host type I interferon production (7, 8). Exposure of naïve CD8 T cells to type I interferons drives rapid acquisition of effector function upon antigenic stimulation (9, 10). The complex interplay between naïve CD8 T cells and type I interferons during bystander activation *early on* following virus exposure remains poorly understood.

Recent advances in immunometabolism firmly suggest that T cell function is closely linked with metabolism (11, 12). Virus exposure as well as type I interferons have been reported to cause metabolic restructuring in immune cells (13, 14). While most studies in the area of T cell immunometabolism have focussed on effector and memory T cell subsets (15, 16), very little is known about the metabolic reprogramming that occurs within naïve CD8 T cells, especially immediately following virus exposure. While the majority of studies on T cell metabolism thus far have captured the roles of the key metabolic pathways glycolysis and oxidative phosphorylation, further investigation of other metabolic pathways and metabolites including NAD^+^ is needed. NAD^+^ is a substrate and redox cofactor for several important metabolic pathways and is required for the function of T cells (17). Intracellular NAD^+^ is an essential cofactor for glycolysis and glycolytic flux is well known to mediate effector vs memory function in T cells (18). Also, the activity of NAD^+^-dependent deacetylase SIRT1 has been shown to promote the formation of memory T cells (19). Further, extracellular NAD^+^ promotes immune response through receptor-mediated downregulation of regulatory T cell populations (20). Similarly, tumor cyclic ADP ribose hydrolase CD38-mediated breakdown of extracellular NAD^+^ promotes T cell exhaustion in tumor microenvironments (21).Therapeutic modulation of NAD^+^ levels has been implemented to control T cell function in conditions like graft versus host disease (GVHD) (22) and experimental allergic encephalomyelitis (EAE) (23, 24). However, the implications for NAD^+^ metabolism in the context of antiviral CD8 T cell immunity remain relatively unknown.

In the present study, using a combination of flow cytometry, quantitative proteomics, metabolomics, and pharmacological inhibition approaches within *in vivo* and *in vitro* settings, we report an NAD^+^ salvage-dependent, type I interferon-driven induction of bystander naïve CD8 T cells (CD8 bT_N_) within 24 hours of viral exposure. Given the importance of bystander activation and the early determinants of antiviral adaptive immunity, this study highlights underappreciated consequences of type I interferon-mediated metabolic reprogramming of naïve CD8 T cells immediately after exposure to viruses.

## Results

### Reovirus exposure drives early induction of CD8 bT_N_ cells *in vivo*

**Figure 1:**
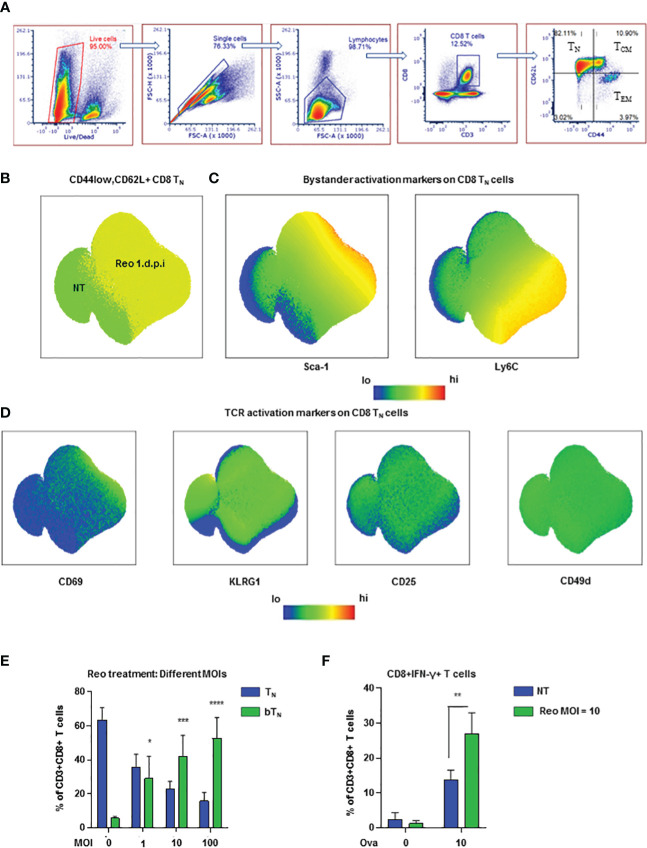
Reovirus induces early bystander activated naïve CD8 T cells *in vivo* **(A)** Representative dot plots demonstrating the gating strategy for the identification of CD8 T cell subsets- T_N,_ T_CM_, T_EM_ and CD44lowCD62Llow (n=3 mice per non-treated group). **(B)** UMAP showing CD8 T_N_ cells in non-treated (NT) and reovirus treated (Reo 1 d.p.i.) C57BL/6 splenocytes. **(C)** UMAP depicting bystander activation markers in CD8 T_N_ cells. **(D)** UMAP representing TCR activation markers in CD8 T_N_ cells. (n=9 mice; NT-3 mice, Reo 1 d.p.i.-6 mice in all UMAP plots). **(E)** Bar graphs for the induction of CD8 bT_N_ cells upon treatment of splenocytes from C57BL/6 mice with varying MOIs of reovirus (n=3 independent experiments). **(F)** Bar graphs for % of CD8+IFN-γ+ T cells in OT-1 splenocytes (n=3 independent experiments). Two-way ANOVA with 95% confidence interval was used for statistical analysis in bar graphs. Significance has been indicated only for CD8 bT_N_ cell populations induced within 24 hours in comparison with untreated control population levels. Not significant (ns) = *p* > 0.05; *p < 0.05; **p < 0.01; ***p < 0.001; **** *p <*0.0001.

To understand the earliest phenotypic changes in CD8 T cells upon virus exposure, we exposed C57BL/6 mice to reovirus, a naturally occurring prototypic dsRNA virus that causes acute infection and drives immune response (25, 26), *via* intraperitoneal (i.p.) injection and studied CD8 T cell subsets *via* flow cytometry in the spleen. We first gated on live cells followed by sequential gating on single cells, lymphocytes and CD3+CD8+ T cells to identify 4 subsets of CD8 T cells- naïve, central memory, effector memory, and CD44lowCD62Llow- using CD44 and CD62L as shown in the representative dot plots (**Figure 1A**). The Ly6 family of proteins including Ly6C and Ly6A/E (Sca-1) are important early indicators of bystander activation (6, 27). We observed that Ly6C was upregulated in CD44lowCD62L+ naïve CD8 T cells within 24 hours of virus exposure (**Figures 1B, C**). Sca-1 was observed to be *uniquely* upregulated on virus exposed naïve CD8 T cells and not in T cells from non-treated spleens and was hence, used as a marker to identify bystander naïve CD8 T cells (CD8 bT_N_) henceforth. Expression of these markers was also observed on the parent CD8 T cell population (**Figures S1A, B**) and it was noted that upregulation in Sca-1 after exposure to virus was not unique to T_N_ cells (**Figure S1C**). Since, CD8 T cells still maintained a naive (CD44lowCD62L+) phenotype, it was unlikely that the increase in Sca-1 and Ly6C expression is due to TCR triggering, however, to confirm this we measured the expression of CD69, CD25, KLRG1, and CD49d (**Figures 1D**, **S1C**), markers known to be upregulated upon activation *via* TCR (6). Naïve CD8 T cells failed to upregulate any of these markers within 24 hours after virus exposure confirming that they were indeed activated in a bystander manner (**Figure 1D**).

Next, we evaluated the presence of CD8 bT_N_ cells over time in different tissues after exposure to reovirus. In the spleen, we found that a significant percentage of CD8 bT_N_ cells were maintained at day 7 after virus exposure- by which timepoint an effector CD8 T cell response was developed (**Figure S2A**). In the mesenteric lymph nodes, CD8 bT_N_ cells were not only maintained but formed the majority population even on day 7 (**Figure S2B**) while in the peritoneal flush extracted from the site of exposure, CD8 bT_N_ cells were also observed albeit at lower percentages (**Figure S2C**). This data demonstrated that CD8 bT_N_ cells were differentially maintained at different sites following virus exposure suggesting that their role and persistence may be context dependent after virus exposure. Since reovirus is a dsRNA virus, we also investigated whether the induction of CD8 bT_N_ cells was dependent on TLR3, a dsRNA sensor in the cytoplasm (28). We discovered that CD8 bT_N_ cell induction was independent of TLR3 (**Figure S2D**). The induction of CD8 bT_N_ cells was also found to be independent of mouse background as a robust CD8 bT_N_ induction was also seen in BALB/c mice after reovirus exposure (**Figure S2E**).

To study CD8 bT_N_ cells *ex vivo*, we exposed mouse splenocytes to varying MoIs of reovirus and observed that they were induced in a dose dependent manner (**Figure 1E**). Upon exposure of OT-1 splenocytes to reovirus for 18h followed by priming with ova peptide for 6h, we observed an increase in the percentage of IFN-γ+ CD8 T cells (**Figure 1F**) suggesting that bystander activation of naïve CD8 T cells led to enhanced functional capabilities. Together, these data show that the exposure to virus prompts the induction of functionally distinct CD8 bT_N_ cells.

### CD8 bT_N_ cells have an innate anti-viral proteomic signature

**Figure 2:**
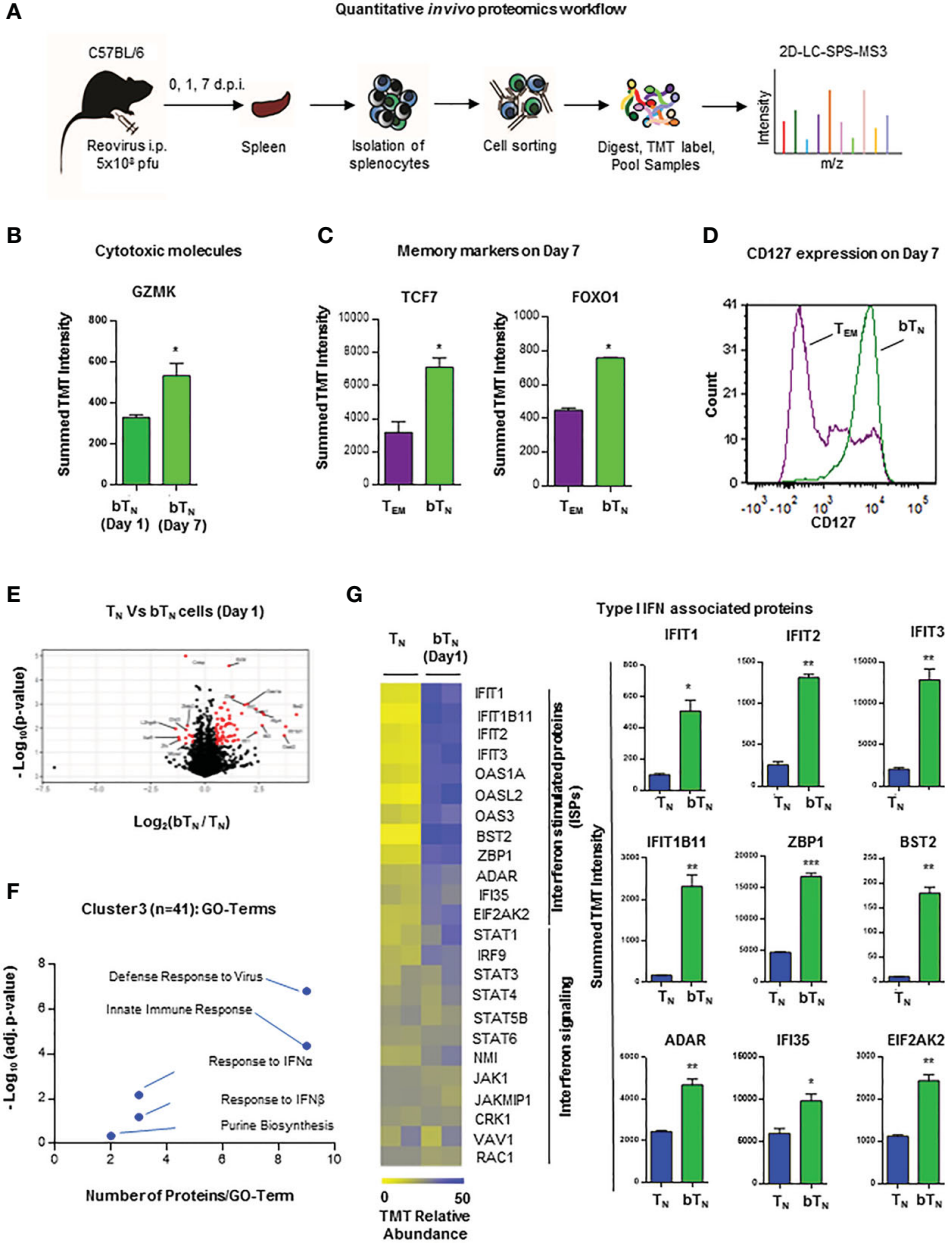
Quantitative *in vivo* proteomic analysis of CD8 bT_N_ cells. **(A)** Schematic for the workflow of quantitative *in vivo* proteomics with T cells isolated from spleens of C57BL/6 mice (n=5 mice pooled for isolation of each cell type in an independent experiment). **(B)** Bar graph for protein expression from quantitative proteomics of granzyme K expression in CD8 bT_N_ cells (1 d.p.i. and 7 d.p.i.). **(C)** Bar graphs for protein expression from quantitative proteomics of memory markers TCF7 and FOXO1 expression in T_EM_ cells (7 d.p.i.) and CD8 bT_N_ cells (7 d.p.i.). **(D)** Histogram overlay for memory marker CD127 in T_EM_ cells (7 d.p.i.) and CD8 bT_N_ cells (7 d.p.i.). Representative histogram shown from spleen of one mouse (n=3 mice) **(E)** Volcano plot compares all identified proteins across CD8 T_N_ and CD8 bT_N_ cells (1 d.p.i.). **(F)** Cluster 3 of GO TERM analysis of proteomics depicting differentially regulated pathways in CD8 bT_N_ cells compared to T_N_ cells. **(G)** Heatmap for the relative levels of interferon-stimulated proteins and interferon signalling proteins identified in T_N_ and CD8 bT_N_ cells (1 d.p.i.). Corresponding bar graphs show the levels of several type I interferon-associated proteins in T_N_ and CD8 bT_N_ cells. Two-tailed Student’s *t*-test with 95% confidence interval was used for statistical analysis. Not significant (ns) = *p* > 0.05; *p < 0.05; **p < 0.01; ***p < 0.001.

In light of phenotypic and functional changes noted above, we next asked whether CD8 bT_N_ cells held a distinct proteomic landscape. For this, a proteomic snapshot of these cells in comparison with other T cell subsets was generated using tandem mass tag (TMT)- labelled multiplexed mass spectrometry. FACS-isolated pure populations of CD8 T_N_ cells, CD8 bT_N_ cells (Day 1), CD8 bT_N_ cells (Day 7) and T_EM_ cells (Day 7) from the spleen were digested, labelled with TMT, and processed for mass spectrometry-based proteome profiling as depicted in the workflow schematic (**Figure 2A**). A heatmap representing 4718 proteins that were identified by the proteomic analysis shows these proteins grouped into various clusters by K-means clustering across the tested T cell subtypes (**Figure S3A**). The top 2 hits in each of the clusters have been listed in **Table S1**. When comparing CD8 bT_N_ cells on Day 1 and Day 7, we discovered that CD8 bT_N_ cells on Day 7 had increased granzyme K expression (**Figure 2B**) although no changes were seen in levels of granzyme A or B (**Figure S3B**). Further, when compared with the other dominant population on Day 7 i.e., T_EM_ cells- CD8 bT_N_ cells had increased expression of memory markers TCF7 and FOXO1 (**Figure 2C**). Using flow cytometry, we also noted that CD8 bT_N_ cells on day 7 were mostly positive for CD127 expression as compared to T_EM_ cells that include distinct CD127+ and CD127- populations (**Figure 2D**) that are known to comprise memory precursors and short-lived effectors, respectively (29–31). Taken together, this data suggests that CD8 bT_N_ cells bear distinct proteome changes accompanying alerted functional capabilities.

To get an insight into the molecular signature of these early induced CD8 bT_N_ cells and understand how they differed from T_N_ cells within 24 hours of virus exposure, we next focussed our analysis on the proteomic data from these two groups of cells, identifying proteins that were significantly changed (at least 2-fold) between them (**Figure 2E**). From among the K-means clusters described earlier, cluster 3 represents proteins that are upregulated in CD8 bT_N_ cells when compared to T_N_ cells. Gene Ontology (GO) term analysis for proteins in this cluster revealed differential expression of proteins related to defense responses to virus, innate immune response, type I interferon, and purine biosynthesis (**Figure 2F**). As mentioned previously, the induction of CD8 bT_N_ cells after reovirus exposure was observed to be independent of TLR3 signalling (**Figure S1B**), however, among the proteins related to defense response to virus and innate immune response, RIG-I related proteins were upregulated in CD8 bT_N_ cells (**Figure S3C**). Along with TLR3, RIG-I is also involved in dsRNA recognition and regulation of immune responses (32). One of the major pathways associated with viral defense, however, identified in CD8 bT_N_ cells *via* proteomics was the type I interferon pathway (**Figure 2E**). Levels of interferon-driven proteins including the IFIT family, ZBP1, BST2, ADR, IFI35 and EIF2AK2 were upregulated in CD8 bT_N_ cells (**Figure 2G**). These data demonstrated a clear signature of type I interferon sensitization, a characteristic event driven during virus recognition, in CD8 bT_N_ cells.

### Antiviral type I interferons induce CD8 bT_N_ cells in a STAT1-dependent manner

**Figure 3:**
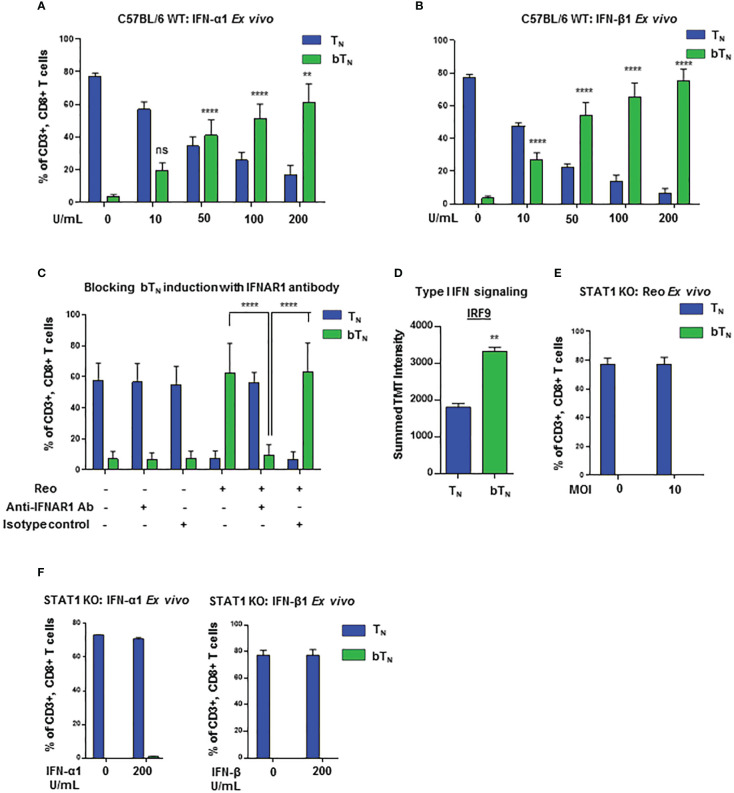
Induction of CD8 bT_N_ cells occurs in a STAT1-dependent manner. Bar graphs showing induction of CD8 bT_N_ cells upon *ex vivo* treatment of splenocytes from C57BL/6 mice with **(A)** varying concentrations of IFN-α1 from 10 U/mL- 200 U/mL (n=3 independent experiments), **(B)** varying concentrations of IFN-β1 from 10 U/mL- 200 U/mL (n=3 independent experiments) and **(C)** reovirus (MOI = 10) + IFNAR1 antibody (10µg/mL, n=3 independent experiments; Significance shown as indicated in figure) or isotype control. **(D)** IRF9 protein intensity (Two-tailed Student’s t-test). **(E, F)** Bar graphs for induction of CD8 bT_N_ cells upon *ex vivo* treatment of splenocytes from STAT1 KO mice with reovirus MoI = 10 (n=3 independent experiments) **(E)** and, IFN-α1 and IFNβ1 (200 units (U)/ml each) (n=2 independent experiements) **(F)**. Two-way ANOVA with Tukey’s multiple comparisons test and 95% confidence interval was used for statistical analysis unless otherwise indicated. Significance has been indicated for CD8 bT_N_ cells in treatment conditions versus non-treated conditions unless otherwise indicated. Not significant (ns) = p > 0.05; **p < 0.01; **** p <0.0001.

In order to further investigate the role of type I interferons in the induction of CD8 bT_N_ cells, we first confirmed that an increased level of type I interferon was observed in the spleen of mice exposed to reovirus (**Figure S3D**). Hence, we next asked if type I interferons might be able to directly drive the induction of CD8 bT_N_ cells without reovirus. To test this, we treated *ex vivo* isolated C57BL/6 splenocytes with exogeneous IFN-α1 and IFN-β1. Both treatments induced a robust, dose-dependent induction of CD8 bT_N_ cells (**Figures 3A, B** respectively). We further tested whether type I interferon signalling had a role to play in the induction of CD8 bT_N_ cells. Using quantitative PCR, we noted that the levels of *Ifnar1* and *Ifnar2* remained unchanged in splenocytes on day 1 after treatment of mice with reovirus (**Figure S3E**). We proceeded to block the IFNAR1 receptor with a blocking antibody during treatment of splenocytes with reovirus *ex vivo*. This led to the abolishment of CD8 bT_N_ cell induction (**Figure 3C**). Within the type I interferon signalling pathway, the ISGF3 (STAT1-STAT2-IRF9) complex forms an important regulator of type I interferon signalling in cells (33). Although signalling proteins in other type I interferon signalling pathways remained unchanged (**Figure 2G**, lower half of heatmap), IRF9 of the ISGF3 complex was significantly upregulated in CD8 bT_N_ cells (**Figure 3D**). In the proteomics data, all STAT proteins, except STAT2, were identified and STAT1 levels were found to be increased, albeit not to statistically significant levels (**Figure S3F**). Nonetheless, given the importance of STATs in the response to type I interferons and the upregulation of IRF9 of the ISGF3 complex in the proteomics data, we decided to test the induction of CD8 bT_N_ cells in STAT1 KO mice. Reovirus failed to induce CD8 bT_N_ cells in splenocytes isolated from STAT1 KO mice (**Figure 3E**). Similar results were also observed with type I interferons wherein no induction of CD8 bT_N_ cells was observed with IFN-α1 and IFN-β1 (**Figure 3F**). In addition, it should be noted that our model of STAT1 KO mice also had a SLAM knock-in. SLAM or CD150 is a receptor for measles virus and this model was previously designed for the study of measles infection (34). To confirm that the lack of induction of CD8 bT_N_ cells in the STAT1 KO mice was not a result of SLAM knock-in, we tested splenocytes from SLAM knock-in only control mice and found robustly induced CD8 bT_N_ cells following treatment with reovirus and IFN-β1 (**Figure S3G**). Altogether, these data conclusively demonstrate a role for type I interferon signalling in the induction of CD8 bT_N_ cells and provide evidence that this induction occurs in a STAT1-dependent manner.

### Induction of CD8 bT_N_ cells with different viruses depends on their interferon activating capacity

**Figure 4:**
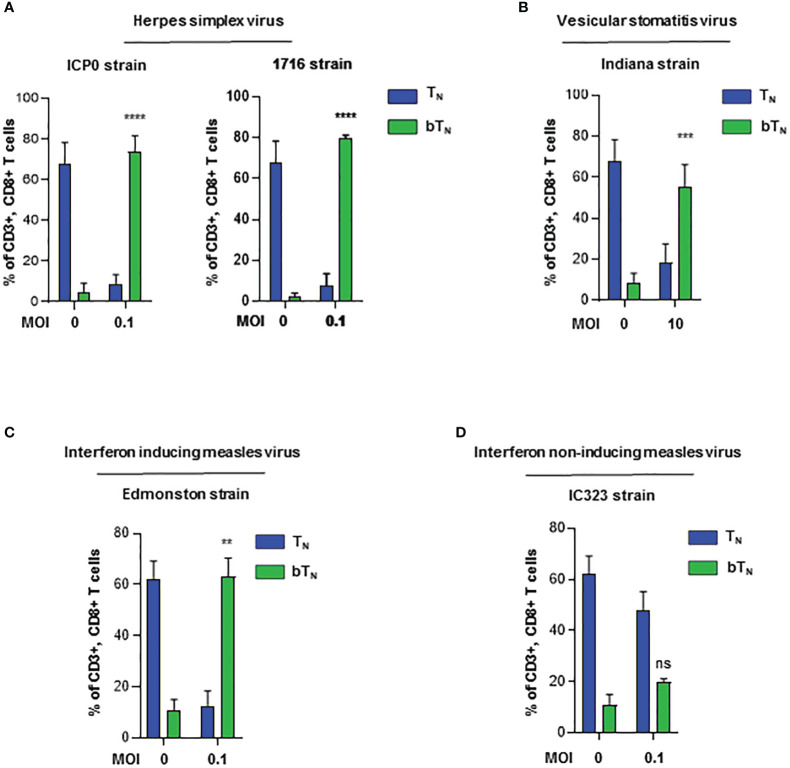
Induction of CD8 bT_N_ cells *ex vivo* upon exposure to different viruses. Bar graphs showing the induction of CD8 bT_N_ cells upon *ex vivo* exposure of C57BL/6 splenocytes to **(A)** two different strains of herpes simplex virus (ICP0 and 1716, n=3 independent experiments each) at MoI = 0.1, **(B)** vesicular stomatitis virus (Indiana strain, n=3 independent experiments) at MoI = 10, **(C)** interferon activating strain of measles (Edmonston, n=2 independent experiments) at MoI = 0.1 and **(D)** wild type strain of measles (IC323, n=2 independent experiments) at MoI = 0.1. Two-way ANOVA with Sidak’s multiple comparisons test and 95% confidence interval was used for statistical analysis in bar graphs. Significance has been indicated only for CD8 bT_N_ cell populations induced within 24 hours in comparison with untreated control population levels. Not significant (ns) = p > 0.05; **p < 0.01; ***p < 0.001; **** p <0.0001.

To assess whether different viruses were equally capable of inducting bystander activation of naïve CD8 T cells, we investigated the induction of CD8 bT_N_ cells upon *ex vivo* exposure to a number of different viruses. We observed that two different strains of herpes simplex virus- HSV- ICP0 (35) and 1716 (36)- robustly induced CD8 bT_N_ cells at a low MoI of 0.1 (**Figure 4A**). Next, the Indiana strain of VSV (37) induced CD8 bT_N_ cells at a higher MoI (**Figure 4B**). Further, we employed measles virus and discovered that the Edmonston vaccine strain of measles (38)- induced CD8 bT_N_ cells at an MoI of 0.1 (**Figure 4C**) however, the IC323 strain of measles virus (39) failed to induce a significant CD8 bT_N_ cell response (**Figure 4D**). Within the array of viruses employed, IC323 strain of measles is known for its inferior capacity to stimulate type I interferons (40, 41) Taken together, this data suggested that CD8 bT_N_ cell induction occurred variably in different viruses and was a function of their interferon inducing capabilities.

### NAMPT-mediated NAD^+^ biosynthesis through salvage pathway metabolism regulates CD8 bT_N_ cell induction

**Figure 5:**
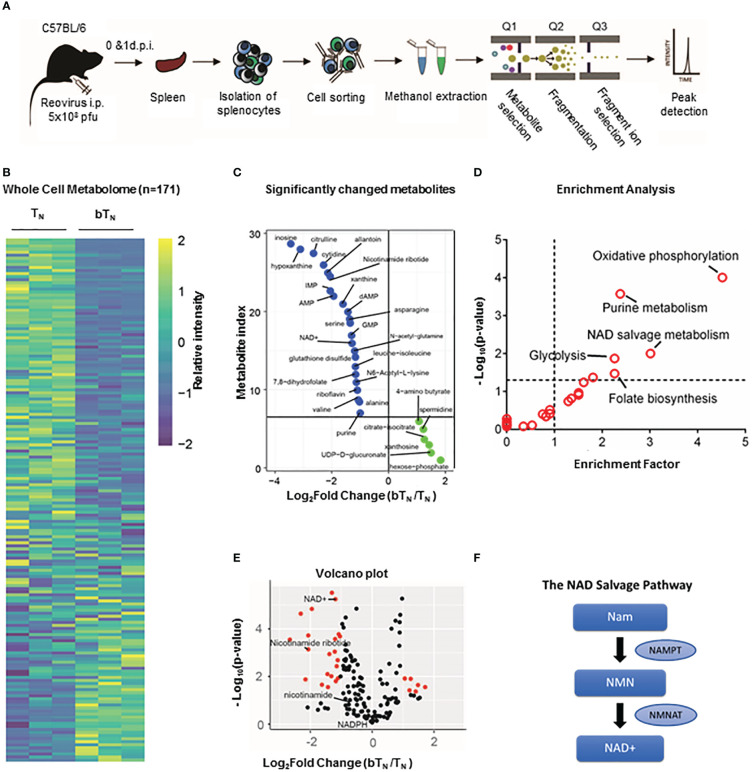
Semi-targeted metabolome analysis of CD8 bT_N_ cells. **(A)** Schematic for the workflow of metabolome analysis of T cells. **(B)** Whole cell metabolome heatmap. **(C)** List of significant top upregulated and downregulated metabolites in CD8 bT_N_ cells. **(D)** Significant metabolite Enrichment analysis of significantly changed metabolites. **(E)** Volcano plot depiciting metabolites that are significantly changed in CD8 bT_N_ cells versus CD8 T_N_ cells. NAD^+^ salvage metabolites are highlighted in red. **(F)** NAD^+^ salvage pathway. NAM- Nicotinamide, NMN- Nicotinamide mononucleotide and NAD^+^- Nicotinamide adenosine dinucleotide.

**Figure 6:**
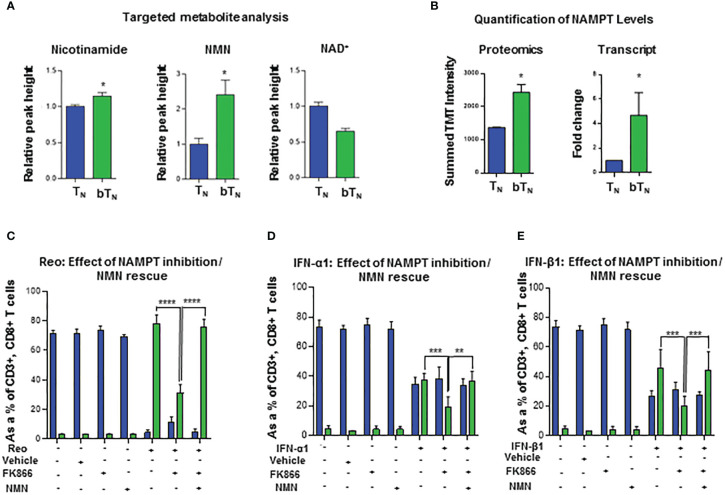
NAD^+^ salvage metabolism regulates induction of CD8 bT_N_ cells. **(A)** Bar graphs depicting the relative peak heights of NAD^+^ salvage metabolites - NAM, NMN and NAD^+^ - using targeted metabolomics (n=4 mice per group). **(B)** Bar graphs for NAMPT levels- proteomics (n=5 mice pooled per condition) and quantitative PCR analysis (n = 3 independent experiments). **(C–E)** Bar graphs show the induction of CD8 bT_N_ cells upon *ex vivo* treatment of splenocytes from C57BL/6 mice with reovirus (MoI = 10) **(C)** IFN-α1 (20 U/mL) **(D)**, and IFN-β1 (20 U/mL) **(E)** in the presence of FK866 (5nM) and NMN (200µM) (n=3 independent experiments for each treatment, ethanol vehicle control for FK866). Two-tailed student t-test used for statistical analysis for **(A, B)** Two-way ANOVA with Tukey’s multiple comaprisons and 95% confidence interval was used for statistical analysis of **(C–E)** Not significant (ns) = p > 0.05; *p < 0.05; **p < 0.01; ***p < 0.001; **** p <0.0001.

Type I interferons have been shown to drive a rewiring of metabolism in a variety of cell types including hepatic cells (42) and innate immune cells (14). In our proteomics analysis, we discovered that proteins related to NAD^+^-dependent ADP-ribosyl transferase activity like PARP9 and PARP14 (**Figure S4A**), as well as many proteins from the OAS family with ATP binding activity were also upregulated (**Figure S4B**) in CD8 bT_N_ cells when compared to T_N_ cells. Consequently, we hypothesized that CD8 bT_N_ cells might be metabolically reprogrammed as compared to CD8 T_N_ cells after reovirus exposure. To test this hypothesis, we employed a semi-targeted approach to study the metabolism of CD8 T_N_ and CD8 bT_N_ cells. CD8 T_N_ cells and CD8 bT_N_ cells (Day 1) were isolated from the spleens of reovirus-injected C57BL/6 mice by flow cytometry and then processed for metabolome analysis (**Figure 5A**). A heatmap comparing the metabolomic signatures of CD8 T_N_ and CD8 bT_N_ cells clearly showed distinct metabolic rewiring of CD8 bT_N_ cells as compared to CD8 T_N_ cells (**Figure 5B**). A list of the top upregulated and downregulated metabolites (fold change greater than or equal to 2, p<0.05) in CD8 bT_N_ cells as compared to T_N_ cells is shown in **Figure 5C**. An enrichment analysis revealed a role for central energy metabolism (glycolysis and oxidative phosphorylation) in CD8 bT_N_ cells (**Figure 5D**). As glycolysis and oxidative phosphorylation have been extensively studied in T cells (12, 43, 44) and both require NAD^+^, we focussed on NAD^+^ metabolism that has shown emergent applications within immune cell biology (21, 22, 45) and was discovered in our analysis (**Figure 5E**). In mammalian cells, the NAD^+^ salvage pathway is a major source for NAD^+^ synthesis (46) and consists of metabolites nicotinamide (NAM), nicotinamide ribotide or nicotinamide mononucleotide (NMN), and NAD^+^ (**Figure 5F**). Using metabolite standards, we conducted a targeted analysis of metabolites involved in the NAD^+^ salvage pathway and found higher relative levels of NAM, NMN, and lower levels of NAD^+^ (normalized to cell number) in CD8 bT_N_ cells compared to CD8 T_N_ cells (**Figure 6A**). Furthermore, our proteomics analysis revealed that Nicotinamide phosphoribosyltransferase (NAMPT), which is the rate limiting enzyme of the NAD^+^ salvage pathway, is increased in CD8 bT_N_ cells (**Figure 6B**). Using qPCR, we found that transcript levels of *Nampt* were also increased in CD8 bT_N_ cells as compared to T_N_ cells (**Figure 6B**). Interestingly, the transcript levels of other enzymes associated with various NAD^+^ pathways (**Figure S4C**) including *Nmnats* (**Figure S4D**) that are part of the salvage pathway as well as other synthesis pathways, enzymes of the *de novo* pathway (**Figure S4E**), and the nicotinamide riboside/nicotinic acid riboside pathway (**Figure S4F**) remained unchanged, indicating a possibly preferential reliance of CD8 bT_N_ cells on NAD^+^ synthesis *via* NAMPT.

To further consolidate our understanding of the role of the NAD^+^ salvage pathway and NAMPT, we tested the effect of FK866, an inhibitor of NAMPT (47, 48), on the induction of CD8 bT_N_ cells upon reovirus exposure. As shown in **Figure 6C**, splenocytes from C57BL/6 mice exposed to reovirus and treated with FK866 showed less induction of CD8 bT_N_ cells compared to those with only reovirus exposure. The robust induction of CD8 bT_N_ cells was rescued when nicotinamide mononucleotide (NMN), a metabolite downstream of the NAMPT enzymatic action, was supplemented in the media (**Figure 6C**). These results showed that FK866 can impair the virus-driven induction of CD8 bT_N_ cells and further consolidated the role of NAMPT within this phenomenon. One possibility was that the reduced induction of CD8 bT_N_ cells could have occurred *via* an indirect effect of FK866 treatment by reducing the production of proinflammatory mediators in splenocytes (23, 49). To elucidate whether FK866 could impair the induction of CD8 bT_N_ cells in the presence of abundant type I interferons, we treated C57BL/6 splenocytes with exogeneous IFN-α1 or IFN-β1 along with FK866 and found impaired induction of CD8 bT_N_ cells compared to splenocytes treated with only interferons (**Figures 6D, E** respectively). Once again, the impaired induction of CD8 bT_N_ cells upon FK866 treatment was rescued by the supplementation of NMN in the media with both IFN-α1 (**Figure 6D**) and IFN-β1 (**Figure 6E**) treatments. In conclusion, these findings suggested that NAD^+^ production *via* NAMPT-mediated salvage pathway is required for the induction of CD8 bT_N_ cells after reovirus or type I interferon exposure.

## Discussion

As is being recognized in the context of the SARS-CoV2 pandemic, the understanding of early immunological events that occur after virus exposure is extremely important. It has become quite clear from various studies on COVID-19 that one the most important factors determining the clinical outcome of the disease is the early induction of type I interferons (7, 8). Early type I interferon responses are associated with mild COVID whereas delayed type I interferon responses leads to poor viral control, delayed and persistent activation of adaptive immunity, and severe COVID (50). In addition, early bystander activation of T cells has also been shown to be an important characteristic of mild disease compared to delayed bystander activation which has been associated with severe disease (51). Bystander activation is one of the earliest ways in which naïve CD8 T cells are activated occurring even before the cells have had an opportunity to be primed with antigen. Most studies focus on the biology of CD8 T cells after antigenic priming or on the bystander activation of pre-existing memory T cells. In this study, we have delineated the molecular mechanisms that govern the induction of early naïve bystander activated CD8 T cells. We demonstrated that CD8 bT_N_ cells are induced and have an anti-viral, type I interferon signature within 24 hours of reovirus exposure, a timepoint that is not typically studied for CD8 T cells. Further, STAT-1 has been demonstrated to play an important role in the maintenance of quiescence in naïve CD8 T cells (52). We demonstrated that the induction of CD8 bT_N_ cells was dependent on STAT-1, an important finding that can provide clues as to the mechanism of differential maintenance of these cells at different sites as observed in our study. We further demonstrated that the induction of CD8 bT_N_ cells was also dependent on the interferon inducing capacity of viruses.

Like our study, another study has investigated bystander activation in naïve CD8 T cells after virus exposure, albeit at later timepoints, and employed Ly6C, another member of the Ly6 family of proteins like Sca-1, to identify these cells (6). The study demonstrated that bystander activated Ly6C+ T_N_ cells had enhanced homing to lymph nodes, improved homeostatic properties and enhanced function. Taken together with this study, ourfindings further highlight the importance of studying early interferon production upon exposure to viruses and provide insight into another avenue through which CD8 T cells can be modulated early on by viruses.

Recent literature on COVID-19 pathobiology has generated an increased appreciation for the role of immunometabolic reprogramming that occurs during virus exposure (53). Viruses can alter the metabolism of cells directly during their replication or *via* the effects of type I interferons (54). For example, type I interferons induce important changes in the metabolism of plasmacytoid dendritic cells by acting on them in an autocrine manner and these changes allow for enhanced immune function (14). Type I interferons can also modulate T cells, especially CD8 T_N_ cells, which are important during virus exposure. Although some studies have investigated the metabolism of T_N_ cells (55), the impact of metabolism in the context of bystander activated CD8 T_N_ cells remains unexplored. In our study we discovered that CD8 bT_N_ cells, despite being phenotypically similar to prototypic T_N_ cells, demonstrate a completely different metabolic signature. In this regard, our findings on the role of the NAD^+^ salvage pathway in CD8 bT_N_ cell induction adds a crucial piece to the metabolic puzzle being investigated. Targeting NAD^+^ salvage metabolism by inhibiting its rate limiting enzyme NAMPT has been previously shown to reduce effector T cell function in the tumor microenvironment (18, 21). In GVHD the functionality of alloreactive T cells was inhibited by targeting NAMPT (22) and similarly, the depletion of NAD^+^ in T cells *via* FK866 treatment reduced demyelination in EAE (23, 24). Studies of metabolism, specifically the NAD^+^ metabolic circuitry in T_N_ cells following activation in native settings by viral exposure, remain few. We have demonstrated a clear role for NAD^+^ salvage metabolism in the reprogramming of bystander activated CD8 T_N_ cells during the acute phase of the immune response, further underscoring the importance of NAD^+^ salvage metabolism in early T cell biology.

Finally, the virus of choice in this study- reovirus- is an oncolytic virus being developed as a cancer immunotherapy agent for the treatment of various tumor types in clinics (56). Induction of type I interferons and bystander naïve CD8 T cells upon exposure to oncolytic viruses can have important consequences for cancer immunotherapy. The role of type I interferons in tumor immunology is widely appreciated (57, 58) and naïve-like T cells with functional capacities have been detected in tumors (59). Further, cancer metabolism can have a direct detrimental impact on T_N_ cells (60). Understanding the impact of oncolytic viruses like reovirus on T_N_ cells *via* bystander activation and consequent metabolic reprogramming represents an important emerging area of research. We believe that the research in this study provides new avenues for immunometabolism research in T cells, specifically CD8 T_N_ cells, and potential therapeutic targets for the reprogramming of T cell immunity. Ultimately, an improved understanding of bystander activated T_N_ cells and immunometabolism following virus exposure will inform fundamental concepts leading to better development of vaccines and treatments against viral infections and for the effective design and development of oncolytic virotherapies.

## Material and methods

### Viruses, cell lines, and reagents

Reovirus (serotype 3, Dearing strain) was cultured, amplified, and isolated using a previously established protocol (61, 62). L929 cells were cultured in minimum essential media (MEM) with (5% vol/vol Glutamax, 5% fetal bovine serum (FBS), 1X sodium pyruvate, 1X nonessential amino acids, and 1X Anti-Anti [Invitrogen, Carlsbad, CA]). Measles viruses (Edmonston and IC323 strains) were obtained from Dr. Christopher Richardson at the Canadian Centre for Vaccinology, Halifax, Nova Scotia. Herpes simplex viruses (ICP0 and 1716) and vesicular stomatitis virus (Indiana strain) was obtained from Dr. Tommy Alain at the University of Ottawa.

### Animal studies

All animal work and *in vivo* experiments were conducted with prior approval from the Ethics Committee at Dalhousie University, Halifax, Nova Scotia. Wild type C57BL/6 and BALB/c mice were purchased from Charles River Laboratory (Montreal, Quebec, Canada). OT-1 transgenic and TLR3 KO mice were obtained from The Jackson Laboratory, United States. SLAM knock-in (KI) mice and SLAM KI, STAT1 KO mice for harvesting splenocytes and *ex vivo* virus treatment were obtained from Dr. Christopher Richardson. Intraperitoneal injections of reovirus (5 x 10^8^ plaque-forming units/ml) were carried out and mice were sacrificed 1, 3 or 7 days post injection to harvest splenocytes for analysis of T cell populations. All animals used for experiments were between the ages of 6-10 weeks.

### Flow cytometry analysis and immune cell sorting

Sample processing for flow cytometry was carried out by harvesting splenocytes in 5mL PBS-EDTA. The cells were filtered using a 40-micron filter and treated with RBC-lysing ammonium chloride (ACK) buffer. The cells were then incubated with CD16/32 Fc blocking antibody in FACS buffer (PBS + 1% EDTA + 1% FBS) for 25 minutes at 4°C, washed and stained with fluorophore labelled primary antibodies in BD Brilliant Stain buffer or FACS buffer for 25 minutes at 4°C. Stained cells were then washed and fixed with 4% paraformaldehyde for 15 minutes. For intracellular staining, fixation/permeabilization of the cells was carried out using the FoxP3/Transcription Factor Staining Buffer Set after staining with extracellular antibodies. Intracellular labelling with IFN-γ antibody in permeabilization buffer was done for 25 minutes at 4°C. The cells were finally washed and resuspended in FACS buffer. Sample data acquisition was done using the BD FACS Symphony A5 or the LSR Fortessa SORP flow cytometers. Data analysis was carried out using BD FACS Diva (BD Bioscience), FCS Express 7 (DeNovo Software, Los Angeles, CA) and FlowJo version 10 (BD biosciences, Ashland, OR). Dimensionality reduction performed on total CD8 T cells and CD8 T cell subsets using the UMAP (version 2.4) FlowJo plugin. Bar graphs were generated using GraphPad (GraphPad Software Inc., San Diego, CA). Anti-mouse BV785 CD45, BV650 CD62L, BV510 CD3, PerCP-Cy5.5 CD8, and APC-H7 CD4 antibodies were purchased from BD Biosciences. FITC CD44, PerCP CD44, BV711 CD44, BV650 CD62L, PE CD62L, PerCP CD8, PE-Cy7 CD3, PE CD3, APC IFN-γ, PE Sca-1, and AF647 Sca-1 were purchased from Biolegend. For cell sorting, splenocytes were harvested and stained *via* the same protocol described for flow cytometry. The paraformaldehyde fixation step was eliminated, and cells were sorted live using FACSAria III, BD Biosciences with a 95-98% purity. The gating strategy for sorting involved gating on lymphocytes (FSC-A vs SSC-A) followed by gating out the doublets. Singlets were then gated for CD3+CD8+ T cells. Following this, CD44 and CD62L expression was observed on CD8+ T cells and CD44lowCD62L+ were further sub-gated as Sca-1+ or Sca-1- to identify and collect bT_N_ and T_N_ cells respectively. In some cases (for proteomic analysis), T_EM_ cells (CD44hiCD62L+) were also identified and collected.

### *Ex vivo* treatment of splenocytes

C57BL/6 mice and others, depending on the experiment, were sacrificed and splenocytes were harvested and prepared using initial steps described for flow cytometry processing. After treatment with ACK buffer for RBC lysis, 3 x 10^6^ cells were plated in 12-well plates for 24 hours and treated with varying MoIs of reovirus, different strains of measles or herpes simplex viruses or vesicular stomatitis virus. For OT-1 mice experiments, splenocytes were isolated as described above and plated in 96-well plates at a concentration of 1-2 x 10^6^ cells/well. These cells were then stimulated with ovalbumin peptide (SIINFEKL) at the 18-hour timepoint for 6 hours. Other *ex vivo* treatments included treatment of splenocytes with varying concentrations of IFN-α1 and IFN-β1. Combination treatments were also carried out such as treatment of splenocytes with reovirus/IFN-β1 and anti-IFNAR1 antibody or isotype control or treatment of splenocytes with reovirus/IFN-α1/IFN-β1 in the presence of FK866 and NMN. For intracellular staining, brefeldin A (2µg/mL) was added to the cells at the 18-hour timepoint for 6 hours. After 24 hours, cells were collected, washed and processed for analysis *via* flow cytometry. Anti-IFNAR1 antibody (catalog no. 127324), isotype control (catalog no. 400198), IFN-α1 (catalog no. 752802) and IFN-β1 (catalog no. 581302) were purchased from Biolegend. FK866 (product no. F8557) and NMN (product no. N3501) and brefeldin A (catalog no. B7651) were purchased from Sigma. Ovalbumin peptide (257-264) was purchased from Genscript (catalog no. RP10611).

### Quantitative *in vivo* proteomics

Animals were injected with reovirus as described above, and T cell subsets were collected either from the naive spleen or spleens on 1 and 7 days post infection (d.p.i.) *via* the cell sorting process described above. Each sample was prepared by pooling spleens of 5 animals for isolation. Isolated cells were washed with PBS, pelleted, and lysed in 6 M guanidine-HCl, 50 mM HEPES, pH 8.5, containing Roche complete mini protease inhibitor mixture (1 tablet per 10 mL) (Roche, Madison, WI). Lysis was performed *via* sonication and cleared by centrifugation. Cysteine residues were reduced using 5 mM dithiothreitol and then alkylated with 14 mM iodoacetamide. Aliquots containing 50 μg of protein were diluted to 1.5 M guanidine-HCl, 50 mM HEPES (pH 8.5) and digested with trypsin (Promega, Madison, WI). Digested peptides were desalted using 60 mg solid-phase C18-extraction cartridges (Waters, Milford, MA), lyophilized, and labelled using TMT 10-plex reagents as described previously (63). Samples were then mixed equally, desalted using solid-phase C18 extraction cartridges (Waters, Milford, MA), and lyophilized. TMT10-labelled samples were fractionated using high-pH reversed phase chromatography performed with an Onyx monolithic 100 × 4.6 mm C18 column (Phenomenex, Torrance, CA). The flow rate was 800 μL/min, and a gradient of 5−40% acetonitrile (10 mM ammonium formate, pH 8) was applied over 60 min using an Agilent 1100 pump (Agilent) from which 12 fractions were collected. Fractions were desalted using homemade Stage Tips, lyophilized, and analyzed with an Orbitrap Fusion mass spectrometer (64) using the SPS-MS3 method (McAlister et al., 2014). Protein identification was performed using a database search against a mouse proteome database (downloaded from UniProtKB in September 2014) concatenated to a mammalian orthoreovirus 3 (Dearing strain) database (downloaded from UniProtKB in September 2014). All false discovery rate (FDR) filtering and protein quantitation was performed as previously described (63). A protein was required to have a minimum total signal-to-noise ratio of 100 in all TMT reporter channels, and the maximum number of missing channels was equal to 8. Data for heat maps and individual protein profiles are represented by relative TMT intensity, which is based on the summed signal-to-noise. The data set was subsequently analyzed *via* K-means clustering with Euclidean distance using MultiExperiment Viewer (MeV) followed by DAVID Bioinformatics Resources (https://david.ncifcrf.gov/) to conduct GO-term analysis for biological processes, molecular functions and cellular compartments on specific clusters. Our total data set was utilized as the background for the data analysis searches. Volcano plot was generated using R statistical analysis package and bar graphs were generated using GraphPad

### *Ex vivo* T cell metabolomics

For metabolomic analysis, each sample was prepared by pooling spleens of 3-5 mice. Live T cell populations were sorted, washed with PBS-EDTA, and resuspended in 100 µl 80% ice cold methanol. 20 µl of methanol extracted cells were combined with 180 µl of HPLC buffer A [95% (vol/vol) water, 5% (vol/vol) acetonitrile, 20 mM ammonium hydroxide, 20 mM ammonium acetate (pH = 9.0)]. The sample was split in triplicates and run using a triple quadrupole mass spectrometer 5500 QTRAP and metabolite levels were analyzed. Based on known Q1 (precursor ion) and Q3 (fragment ion) transitions, the metabolite name, the dwell time and the appropriate collision energies (CEs) for both positive and negative ion modes were identified. Using this protocol, a selected reaction monitoring transition list of 289 (approximately 10–14 scans per metabolite peak) metabolites can be accurately identified. MultiQuant v2.0 software was used to integrate the peak areas from the Q3 TIC values across the chromatographic elution. Each metabolite from every sample was manually confirmed; typically, a single dominant peak will be present for most detectable compounds. Peak heights normalized to the sum of peak heights per sample were used to determine relative metabolite concentrations between samples. For computational analysis, the metabolomics database was analyzed using R, to identify metabolites with significant fold changes and p values. Metabolite rank was obtained by comparing significantly changing metabolites in the order of decreasing fold change (CD8 bT_N_/CD8 T_N_). NAM, NMN, and NAD^+^ were further analyzed using a targeted analysis on a QExactive Orbitrap Mass Spectrometer. Here, a targeted selective ion monitoring (tSIM) method was employed with a resolution of 140,000. Metabolic features identified by tSIM were confirmed using Maven software and peak heights were exported to excel. Peak heights were again normalized using cell number and relative peak heights calculated. Graphs were generated GraphPad Prism.

### Enrichment analysis

All detectable metabolites were organized into specific and general metabolic pathways based on Kyoto Encyclopedia of Genes and Genomes (KEGG) metabolic pathways. To determine pathways that were enriched in CD8 bT_N_ cells, an overrepresentation analysis was completed. Briefly, metabolites that were increased or decreased by 2-fold and t test probability less than or equal to 0.05 were selected, and compared with the original list of metabolites, and then we calculated percent of metabolites changed. We used this percentage to calculate the expected number of metabolites that would change if the metabolites were randomly distributed throughout the various metabolic pathways. We then calculated fold enrichment by dividing metabolites changed by 2-fold by the number of metabolites expected to have changed if random. Probability was calculated by the hypergeometric test.

### Quantitative real-time PCR

T cells were sorted from splenocytes using flow cytometry and used for RNA extraction and qPCR. 3-5 animals were pooled for isolation of every sample, and this was repeated 2-3 times. RNA extractions were conducted using standard TRIzol methodology following manufacturer’s instructions (Qiagen). Extracted RNA was quantified, diluted to a total of 2 μg, and synthesized into cDNA using Superscript II (Invitrogen, Burlington, ON). SsoAdvanced Universal SYBR Green Supermix (Biorad catalog no. 1708882) was used for qPCR and run on the BioRad CFX96 mice for amplification and quantification. Gene-specific primers for murine *Ifna2*, *Ifnr1*, *Ifnr2*, *Nampt, Kynu, Kyat1, Qprt, Nmrk1, Nmnat1, Nmnat2, Nmnat3, Gapdh* and *Hprt* were purchased from Invitrogen (**Table S2**). The data from the qPCR were collected and analyzed using Livak and Schmittgen’s 2^-ΔΔCT^ method (65). The fold change was calculated by first normalizing the quantification cycle (Cq) of the indicated gene against *Gapdh* or *Hprt* followed by a comparison against the respective controls and bar graphs were generated using GraphPad.

### Statistical analysis

Depending on the indicated experiment, two-way ANOVA with Sidak/Tukey’s post-test or a two-tailed Student’s *t*-test with 95% confidence interval were used for statistical analysis, and *p* values of <0.05 were considered significant. Asterisks were used to signify *p* values as follows: not significant (ns) = *p* > 0.05; *p < 0.05; **p < 0.01; ***p < 0.001; **** *p <*0.0001.

## Data availability statement

The original contributions presented in the study are publicly available. This data can be found here​​​​​​​: PRIDE database - https://www.ebi.ac.uk/pride/. accession: PXD039614.

## Ethics statement

The animal study was reviewed and approved by University Committee on Laboratory Animals (UCLA), Dalhousie University.

## Author contributions

Conceptualization, NH and SG. Methodology, NH, BK, JM, GS, JP. Software, NH, BK, JM, JP, PK, MG, TB-C. Validation, NH, BK, ME. Formal Analysis, NH, BK, JM, PK, MG TB-C. Investigation, NH, BK, JM, ME, VK, YK, GS, JP. Writing- Original Draft, NH. Writing- Review & Editing, YK, BK, GS. Visualization, NH, BK, PK, MG, TB-C. Supervision, StG, CR, SG. Project Administration, NH, SG. All authors contributed to the article and approved the submitted version.
