# Supplementary material for: After virus exposure, early bystander naïve CD8 T cell activation relies on NAD+ salvage metabolism
Source: Front Immunol. 2023 Feb 1;13:1047661. doi: 10.3389/fimmu.2022.1047661 (PMC9932030; doi:10.3389/fimmu.2022.1047661)
Supplement: Supplementary file 1 [file DataSheet_1.pdf]

Figure S1.

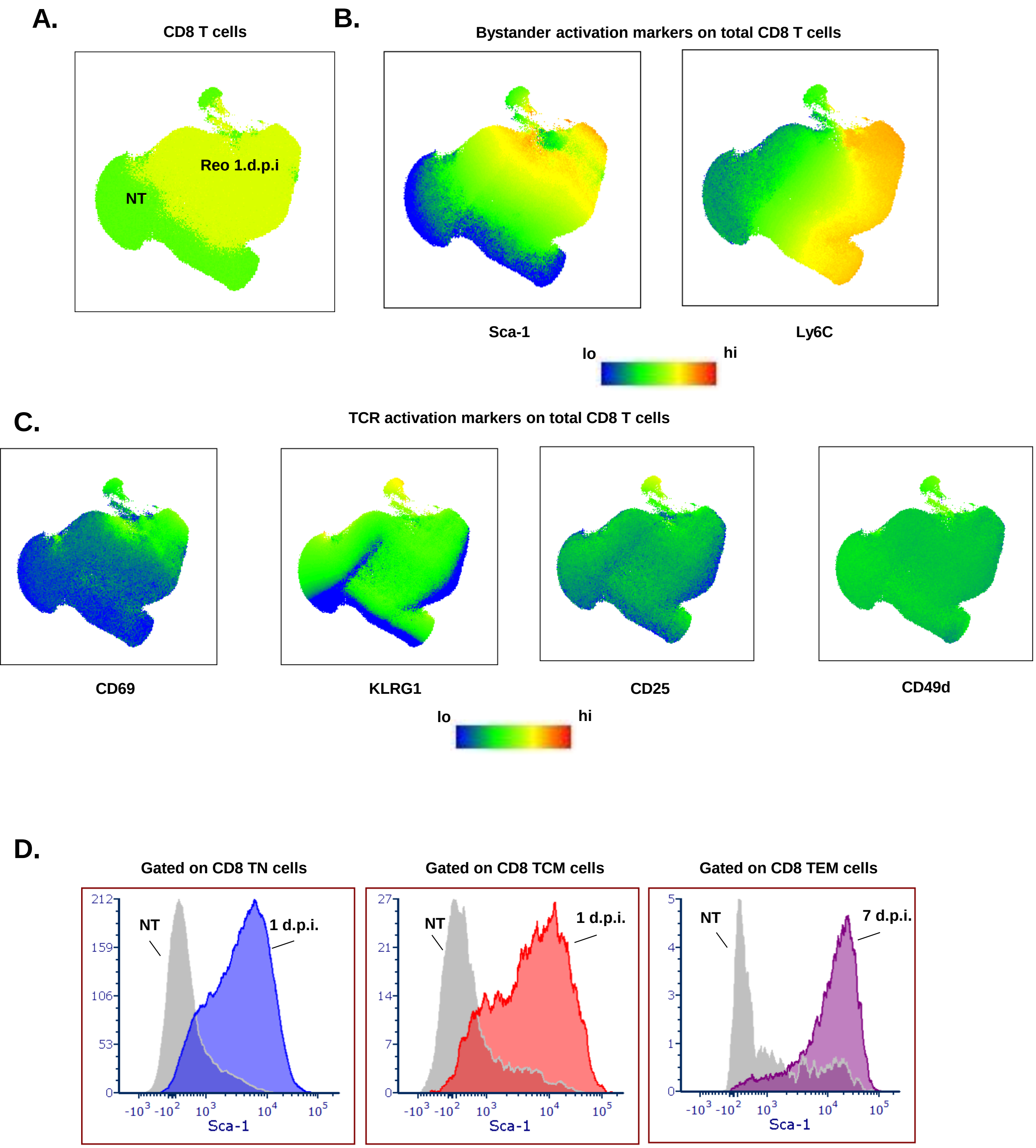
A.
CD8 T cells
Reo 1.d.p.i
NT
B.
Bystander activation markers on total CD8 T cells
Sca-1
Ly6C
lo
hi
C.
TCR activation markers on total CD8 T cells
CD69
KLRG1
CD25
CD49d
lo
hi
D.
Gated on CD8 TN cells
Gated on CD8 TCM cells
Gated on CD8 TEM cells
NT
1 d.p.i.
NT
1 d.p.i.
NT
7 d.p.i.
212
159
106
53
0
27
21
14
7
0
5
4
3
1
0
-10^3 -10^2 10^3 10^4 10^5
Sca-1

Figure S2.

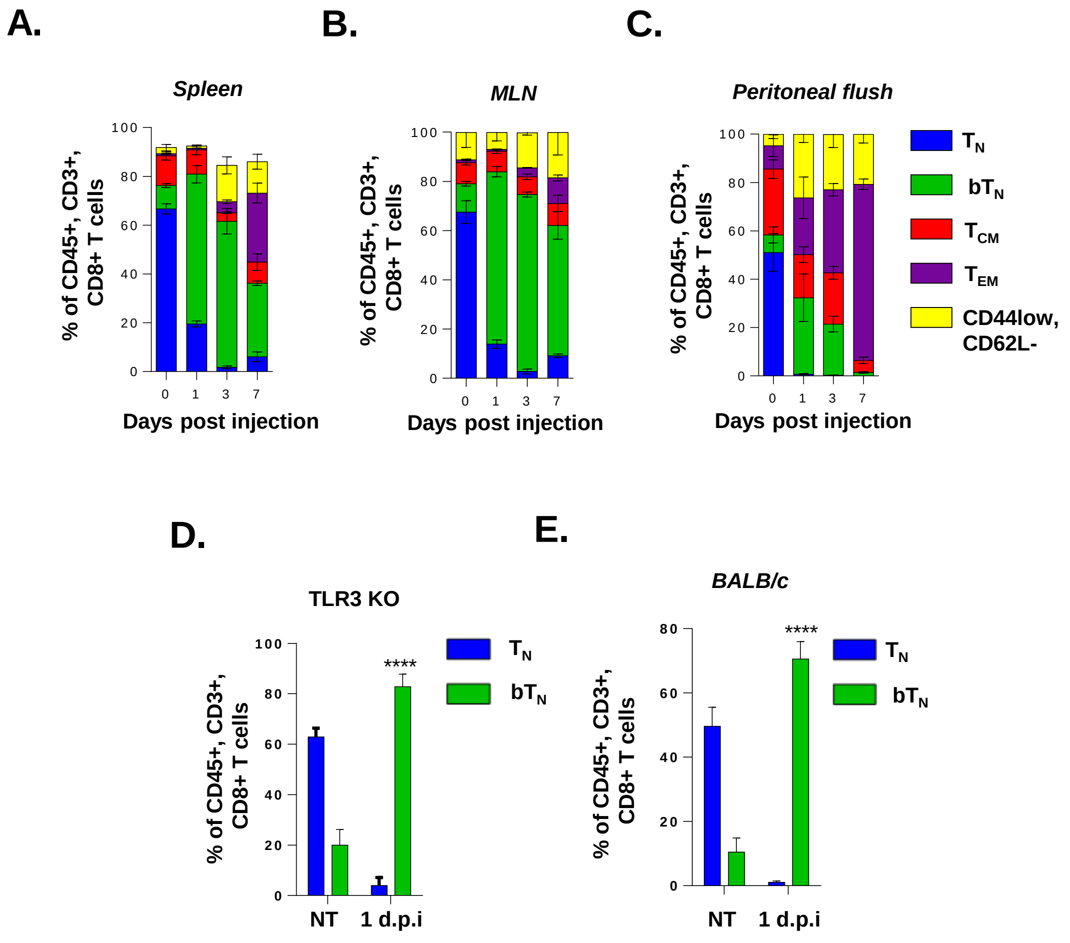
A.
Spleen
% of CD45+, CD3+, CD8+ T cells
Days post injection
B.
MLN
% of CD45+, CD3+, CD8+ T cells
Days post injection
C.
Peritoneal flush
% of CD45+, CD3+, CD8+ T cells
Days post injection
T_N
bT_N
T_{CM}
T_{EM}
CD44low, CD62L-
D.
TLR3 KO
% of CD45+, CD3+, CD8+ T cells
****
NT
1 d.p.i
T_N
bT_N
E.
BALB/c
% of CD45+, CD3+, CD8+ T cells
****
NT
1 d.p.i
T_N
bT_N

Figure S3.

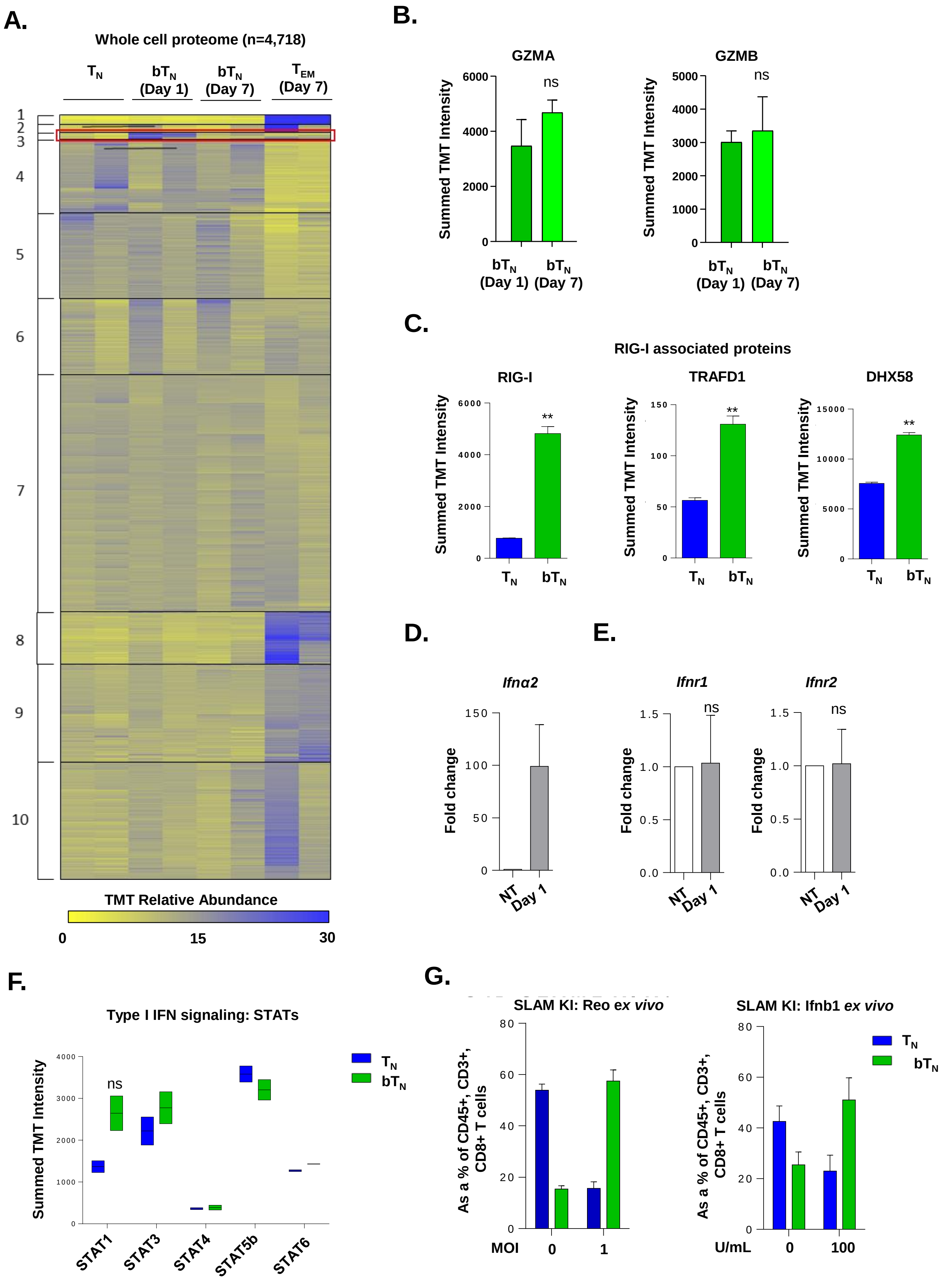

A.
Whole cell proteome (n=4,718)
T_N
bT_N (Day 1)
bT_N (Day 7)
T_EM (Day 7)
1
2
3
4
5
6
7
8
9
10
TMT Relative Abundance
0
15
30
B.
GZMA
GZMB
Summed TMT Intensity
ns
0
2000
4000
6000
1000
3000
5000
bT_N (Day 1)
bT_N (Day 7)
C.
RIG-I associated proteins
RIG-I
TRAFD1
DHX58
**
T_N
bT_N
0
50
100
150
5000
10000
15000
D.
E.
Ifnα2
Ifnr1
Ifnr2
Fold change
0.0
0.5
1.0
1.5
NT
Day 1
F.
Type I IFN signaling: STATs
STAT1
STAT3
STAT4
STAT5b
STAT6
G.
SLAM KI: Reo ex vivo
SLAM KI: Ifnb1 ex vivo
As a % of CD45+, CD3+, CD8+ T cells
MOI
0
1
U/mL
100
20
40
60
80

Figure S4.

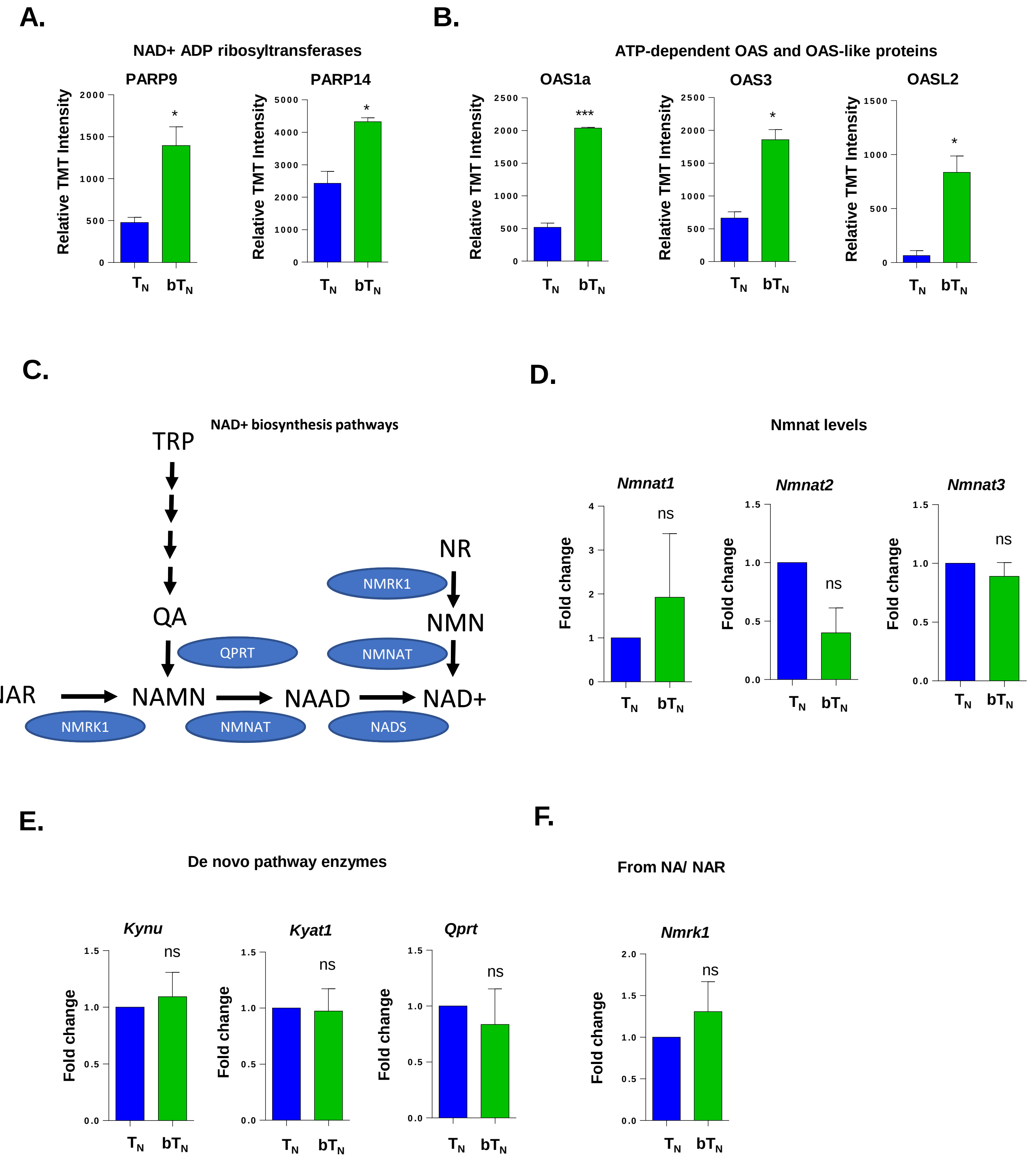

A.
NAD+ ADP ribosyltransferases
PARP9
PARP14
Relative TMT Intensity
T_N
bT_N
*
B.
ATP-dependent OAS and OAS-like proteins
OAS1a
OAS3
OASL2
***
C.
NAD+ biosynthesis pathways
TRP
QA
QPRT
NR
NMRK1
NMN
NMNAT
NAMN
NAAD
NAD+
NADS
D.
Nmnat levels
Nmnat1
Nmnat2
Nmnat3
Fold change
ns
E.
De novo pathway enzymes
Kynu
Kyat1
Qprt
F.
From NA/ NAR
Nmrk1
