# Supplementary material for: After virus exposure, early bystander naïve CD8 T cell activation relies on NAD+ salvage metabolism
Source: Front Immunol. 2023 Feb 1;13:1047661. doi: 10.3389/fimmu.2022.1047661 (PMC9932030; doi:10.3389/fimmu.2022.1047661)
Supplement: Supplementary file 2 [file DataSheet_2.docx]

**Figure S1: A.** UMAP projection of CD8 T cells in non-treated (NT) and reovirus treated (Reo 1 d.p.i.) C57BL/6 splenocytes. **B.** UMAP depicting bystander activation markers in CD8 T cells. **C.** UMAP representing TCR activation markers in CD8 T cells. (n=9; NT-3, Reo 1 d.p.i.-6 in all UMAP plots). **C.** Representative overlay histograms showing the expression of Sca-1 in T_N_ and T_CM_ cells in NT and 1 d.p.i. splenocytes and in T_EM_ cells in NT and 7 d.p.i. splenocytes after reovirus exposure.

**Figure S2:** Time course for induction of CD8 bT_N_ cells at multiple sites in C57BL/6 mice (n=3 each)- A. Spleen. **B.** Mesenteric lymph node. **C.** Peritoneal flush. D. Bar graphs showing the induction of CD8 bT_N_ cells *in vivo* in TLR3 KO mice (n=3-5) within 24 hours of reovirus exposure. E. Bar graphs for the induction of CD8 bT_N_ cells in BALB/c mice within 24 hours of reovirus exposure (n=3). 2-way ANOVA with significance shown for CD8 bT_N_ cells 24 hours after virus exposure compared to same population in non-treated mice controls. Not significant (ns) = p > 0.05; *p < 0.05; **p < 0.01; ***p < 0.001; **** p <0.0001.

**Figure S3:** **A.** Heatmap for the whole cell proteome of 4 different subsets of T cells. Naïve CD8 T cells- T_N_, bystander naïve CD8 T cells 1 day post injection- CD8 bT_N_ (1 d.p.i.), bystander naïve CD8 T cells 7 days post injection- CD8 bT_N_ (7 d.p.i.) and effector CD8 T cells 7 days post injection- T_EM_ (7 d.p.i.). **B.** Bar graphs depicting the levels of Granzyme A and B in bT_N_ cells on 1 d.p.i and 7 d.p.i. **C.** Bar graphs for RIG-I associated proteins in CD8 T_N_ and CD8 bT_N_ cells (1 d.p.i.). Quantitative PCR analysis of **D..** *Ifnα2*, **E..** *Ifnr1* and *Ifnr2* levels in splenocytes of non-treated (NT) mice and mice treated with reovirus 1 d.p.i. **F.** Protein levels of STAT proteins in CD8 T_N_ and CD8 bT_N_ cells from quantitative *in vivo* proteomics analysis. **G.** Bar graphs for the induction of CD8 bT_N_ cells upon treatment of splenocytes of SLAM knock-in (KI) mice with reovirus and IFN-β1 *ex vivo*. Two-tailed student t-test used for statistical analysis of **A-F.** Not significant (ns) = p > 0.05; *p < 0.05; **p < 0.01; ***p < 0.001; **** p <0.0001.

**Figure S4:** Bar graphs for protein levels of **A.** NAD^+^- dependent ribosyl transferases and **B.** ATP-dependent OAS and OAS-like proteins in CD8 T_N_ and CD8 bT_N_ cells (1 d.p.i.) from quantitative *in vivo* proteomics analysis. **C.** Bar graphs for transcript levels of NMNATs. **D.** NAD^+^ biosynthesis pathways. TRP- tryptophan, QA- quinolinic acid, NR- nicotinamide riboside, NMN- nicotinamide mononucleotide, NAD^+^- nicotinamide adenine dinucleotide, NAR- nicotinic acid riboside, NAMN- nicotinic acid mononucleotide and NAAD- nicotinic acid adenine dinucleotide. Bar graphs for transcript levels of **E.** *de novo* pathway enzymes and **F.** *Nmrk1* for synthesis from NR/ NAR. Two-tailed student t-test used for statistical analysis. Not significant (ns) = p > 0.05; *p < 0.05; **p < 0.01; ***p < 0.001; **** p <0.0001.

**Table 1. K-means clustering summary**

| **Cluster:** | **Gene-Ontology: Biological Function** | **Proteins/ Term** | **Benjamini Value:** |
| --- | --- | --- | --- |
| 1 | DNA Replication | 8 | 5.20E-05 |
|  | Cell Cycle | 13 | 9.20E-05 |
| 2 | Protein Transport | 7 | 9.90E-01 |
|  | Endocytosis | 4 | 9.80E-01 |
| 3 | Defense Response to Virus | 9 | 1.50E-07 |
|  | Innate Immune System | 9 | 4.20E-05 |
| 4 | rRNA Processing | 23 | 4.60E-11 |
|  | Ribosome Biogenesis | 17 | 5.00E-08 |
| 5 | Oxidation-Reduction Process | 51 | 3.10E-08 |
|  | Metabolic Process | 35 | 3.90E-05 |
| 6 | Antigen Processing and Presentation via MHC Class I | 12 | 4.30E-08 |
|  | Proteolysis Involved in Cellular Protein Catabolism | 12 | 4.20E-05 |
| 7 | mRNA Processing | 119 | 9.70E-51 |
|  | RNA Splicing | 97 | 9.70E-45 |
| 8 | DNA Replication | 14 | 4.60E-05 |
|  | Cell Cycle | 29 | 7.70E-05 |
| 9 | Translation | 45 | 4.50E-11 |
|  | Protein Folding | 23 | 1.50E-08 |
| 10 | Protein Transport | 66 | 3.40E-14 |
|  | Translation Initiation | 14 | 1.40E-05 |

**Table 2. qPCR primer sequences**

| **Gene name** | **Primer sequence** | **NCBI Accession** |
| --- | --- | --- |
| *Ifnα2* | F: CTGTGCTTTCCTCGTGATGCT  R: AAAGGGGAGCCTCCTCATCTG | NM_010503.2 |
| *Ifnr1* | F: GGTCATTACTGTCACCGCCA  R: ACACAGTACACAGTCAGCGG | NM_010508.2 |
| *Ifnr2* | F: AGAGAACAAGTCTGGCCCAC  R: CACTATGACGATGGCGACGA | NM_010509.2 |
| *Nampt* | F: ATGGTCATCTCCCGATTGAAGTAAA  R: TTGGATACCAGGACTGAACAAGAAT | NM_021524.2 |
| *Kynu* | F: CCTGCGTTAGTGGGATGGTT  R: AGACCTCTAAACTGGCGTGC | NM_027552.3 |
| *Kyat1* | F: ATCTAGGGCTCGGCCTCTTT  R: CCTGAGTCCCACACACAGTT | NM_172404.3 |
| *Qprt* | F: ACTGTGGTGAACACACTGAC  R: GTTGTAGGGGGTAACAGGAGC | BC011191.1 |
| *Nmrk1* | F: GTGTGATTTCCAAAGCCAGTTATGA  R: GGCTTGAAGAAGTCATCCTGAGATA | NM_145497.2 |
| *Nmnat1* | F: ACAATGGCTGGGCCTTTAGA  R: ACATGCAACCCTCTGACAGC | NM_001356357.1 |
| *Nmnat2* | F: TGTTCGAGAGAGCCAGGGAT  R: GTGTCCTGATAGCACTCCCAT | BC089007.1 |
| *Nmnat3* | F: ACGACAGAGTCAGATGGCTG  R: ATATTCGTGATGGGGTTGAAGGAAC | NM_144533.3 |
| *Gapdh* | F: GAGAGTGTTTCCTCGTCCCG  R: ATGAAGGGGTCGTTGATGGC | NM_001289726.1 |
| *Hprt* | F: CAGTCCCAGCGTCGTGATTA  R: TGGCCTCCCATCTCCTTCAT | NM_013556.2 |
